# Epigallocatechin-3-gallate alleviates gestational stress-induced postpartum anxiety and depression-like behaviors in mice by downregulating semaphorin3A and promoting GSK3β phosphorylation in the hippocampus

**DOI:** 10.3389/fnmol.2022.1109458

**Published:** 2023-01-26

**Authors:** Fang Xu, Hui Wu, Linghua Xie, Qing Chen, Qi Xu, Lihong Sun, Hua Li, Jiaqian Xie, Xinzhong Chen

**Affiliations:** Department of Anesthesia, Women’s Hospital, Zhejiang University School of Medicine, Hangzhou, China

**Keywords:** postpartum depression, gestational stress, epigallocatechin-3-gallate, Sema3A, GSK3β

## Abstract

**Introduction:**

Postpartum depression (PPD) is a common neuropsychiatric disorder characterized by depression and comorbid anxiety during the postpartum period. PPD is difficult to treat because of its elusive mechanisms. Epigallocatechin-3-gallate (EGCG), a component of tea polyphenols, is reported to exert neuroprotective effects in emotional disorders by reducing inflammation and apoptosis. However, the effect of EGCG on PPD and the underlying mechanism are unknown.

**Methods:**

We used a mouse model of PPD established by exposing pregnant mice to gestational stress. Open field, forced swimming and tail suspension tests were performed to investigate the anxiety and depression-like behaviors. Immunohistochemical staining was used to measure the c-fos positive cells. The transcriptional levels of hippocampal semaphorin3A(sema3A), (glycogen synthase kinase 3-beta)GSK3β and collapsin response mediator protein 2(CRMP2) were assessed by RT-PCR. Alterations in protein expression of Sema3A, GSK3β, p-GSK3β, CRMP2 and p-CRMP2 were quantified by western blotting. EGCG was administrated to analyze its effect on PPD mice.

**Results:**

Gestational stress induced anxiety and depression-like behaviors during the postpartum period, increasing Sema3A expression while decreasing that of phosphorylated GSK3β as well as c-Fos in the hippocampus. These effects were reversed by systemic administration of EGCG.

**Conclusions:**

Thus, EGCG may alleviate anxiety and depression-like behaviors in mice by downregulating Sema3A and increasing GSK3β phosphorylation in the hippocampus, and has potential application in the treatment of PPD.

## 1. Introduction

Depression is a major contributor to the global burden of disease (Galea and Frokjaer, 2019) and affects twice as many women as men. The highest rates of anxiety and depression in a woman’s lifetime are during the postpartum period (Pawluski et al., 2017). Postpartum depression (PPD) is one of the most common complications of childbirth, affecting 14% of women (Liu et al., 2022) and negatively impacting infant behavior, mood, and cognitive development (O’Hara and McCabe, 2013). Despite the high incidence of PPD and its debilitating consequences for mothers and children, little is known about the etiology of PPD although stress is a major risk factor (Sandman et al., 2012) that is shown to be associated with ~90% of cases in a European population (Qiu et al., 2020). Women who experience chronic psychosocial stress during the perinatal period are susceptible to PPD (Robertson et al., 2004), while gestational stress during the postpartum period is shown to increase anxiety and depression-like behaviors in rodents (Hillerer et al., 2011; Tan et al., 2018; Zoubovsky et al., 2020). Elucidating the behavioral, physiological, and molecular changes caused by stress during pregnancy could provide insight into the mechanisms underlying the development of PPD.

Semaphorin 3A (Sema3A) is a secreted protein in the nervous system that is involved in axon guidance (Perez et al., 2021), neuronal migration (Chen et al., 2008), and synapse formation (Tillo et al., 2012). Sema3A has been implicated in neuropsychiatric diseases such as schizophrenia (Gilabert-Juan et al., 2015), Alzheimer’s disease (AD; Wang et al., 2021) and epilepsy (Van Battum et al., 2015), and its accumulation in hippocampal neurons induces programmed cell death, which is shown to contribute to AD development (Good et al., 2004). An elevated level of Sema3A in the cerebellum of patients with schizophrenia is associated with the downregulation of genes related to the formation and maintenance of synapses, thereby reducing synaptic plasticity (Eastwood et al., 2003). Additionally, variants of the *Sema3A* gene are found to be linked to comorbid alcohol dependence and major depression (Zhou et al., 2017), suggesting a role for Sema3A in the etiology of depression.

Glycogen synthase kinase 3β (GSK3β) is a serine/threonine protein kinase and downstream target of Sema3A (Williams et al., 2004) that participates in numerous physiologic processes such as cell cycle regulation and synaptic transmission and plasticity (Duda et al., 2020). GSK3β also plays a role in major depression; it is a target of mood stabilizers and antidepressants (Li and Jope, 2010), and selective GSK3β inhibitors are shown to exert antidepressant action in rodents (Gould et al., 1999; Kaidanovich-Beilin et al., 2004). Collapsin response mediator protein 2 (CRMP2) is a phosphorylation target of GSK3β (Wilson et al., 2014; Zhang and Koch, 2017) that has been implicated in schizophrenia (Zhang et al., 2016), AD (Yang et al., 2017) and depression (Xiang et al., 2020).

Epigallocatechin-3-gallate (EGCG) is a catechin polyphenol compound found in tea that has anti-inflammatory, antioxidative, and neuroprotective effects in mammals and may have therapeutic value for the treatment of various diseases (Chakrawarti et al., 2016). In male rats, EGCG shows a protective effect against chronic stress-induced depression (Li G. et al., 2020) and alleviates anxiety-like behavior by inhibiting neuroinflammation and apoptosis in the hippocampus (Wang et al., 2020).

Although Sema3A, GSK3β, and CRMP2 have been linked to depression, their role in PPD has not been reported. Moreover, the therapeutic potential of EGCG for the treatment of PDD is not known. Meanwhile, the hippocampus is considered as a vital brain region related to PPD in both basic and clinical research (Pawluski et al., 2017). The hippocampus contains high levels of glucocorticoid receptors and regulates the hypothalamus-pituitary–adrenal (HPA) axis, making it more susceptible to stress and depression. In this study, we employed real-time PCR, western blotting and behavioral tests to study the effect of EGCG in PPD. C-Fos, a robust indicator for neuronal activity, is generally induced by a variety of stimulation (Joo et al., 2016). Therefore, we used c-Fos staining to detect the activity of hippocampal neurons. We found that gestational stress induced anxiety and depressive-like behaviors, and decreased c-Fos expression in hippocampus during the postpartum period. Importantly, EGCG alleviated gestational stress-induced postpartum anxiety and depression symptoms, which was associated with the downregulation of Sema3A and increase of phosphorylated GSK3β in the hippocampus. These results suggest that EGCG may be an effective treatment for PPD induced by gestational stress.

## 2. Materials and methods

### 2.1. Animals

Pregnant C57BL/6 mice at gestational day 7 (G7) were purchased from Ziyuan Experimental Animal Technology Co. (Hangzhou, China). The mice were housed in a humidity-and temperature-controlled animal room with free access to food and water. The room was maintained on a standard 12:12-h light/dark cycle, with lights on at 07:00 and off at 19:00. The animals were treated in accordance with protocols approved by the Animal Ethics and Welfare Committee of Zhejiang University School of Medicine. All experimental procedures were carried out in accordance with the National Institutes of Health (NIH) Guide for Care and Use of Laboratory Animals (Publication no. 86–23).

### 2.2. Animal model and groups

**Figure 1:**
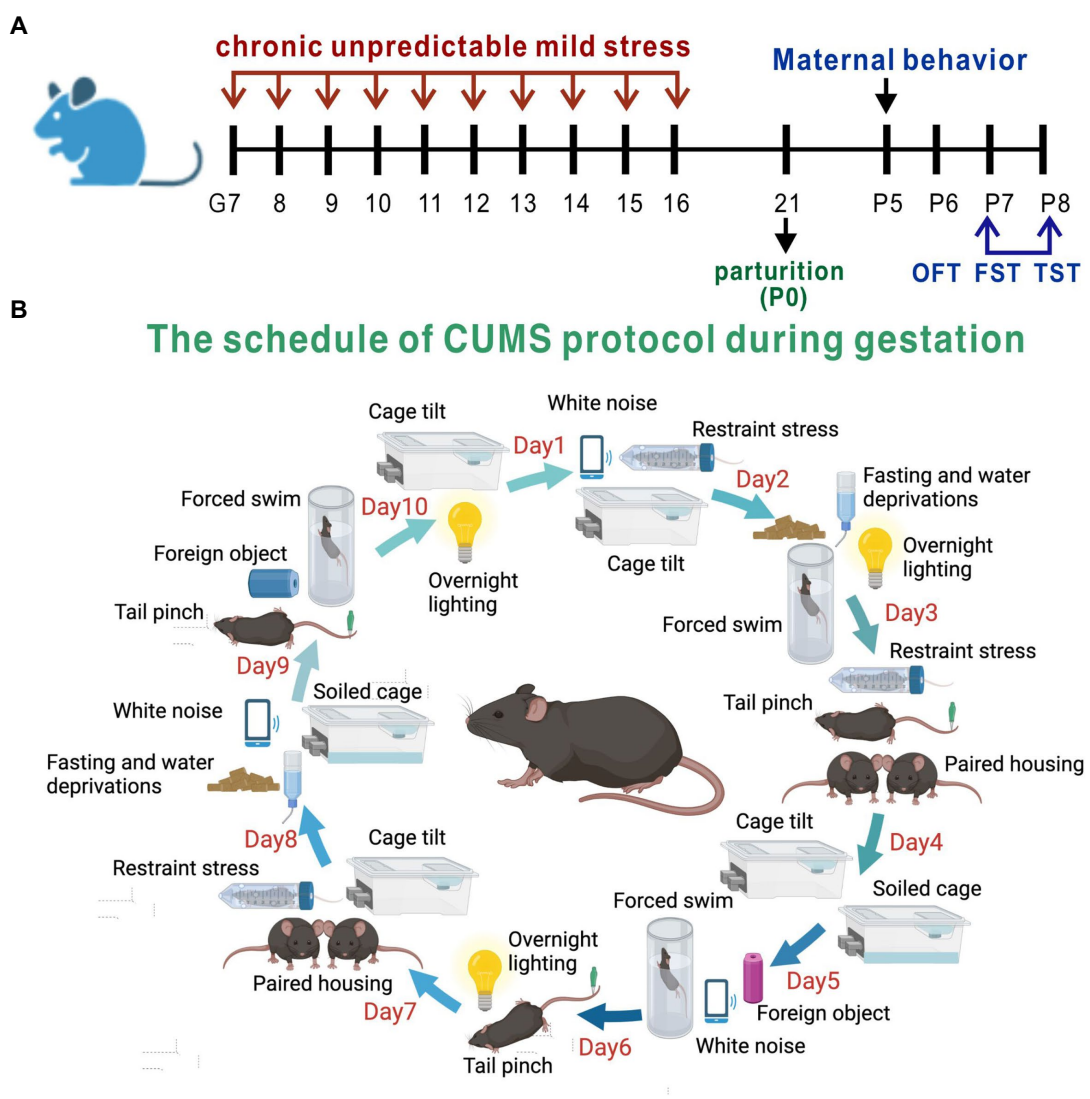
Experimental design and the method of PPD model establishment. **(A)** The timeline of model establishment and behavioral tests. **(B)** The schedule of CUMS protocol during gestation.

Pregnant mice were randomized into a PPD group, a PPD + EGCG group and a control group. The gestational stress-induced PDD model was established as previously described (Kiryanova et al., 2016), with some modifications. From G7 to G16, mice were subjected to a regimen of chronic unpredictable mild stress (CUMS; Table 1; Figure 1B). Stressors included restraint stress (mice were restrained in conical tubes with holes for airflow), white noise, cage tilting (home cage tilted by 45°), fasting and water deprivation, forced swim (5 min), overnight lighting, tail pinching (2 min), housing in pairs (mice were paired with a pregnant dam in their home cage or in the pregnant dam’s cage), soiled cage (with 100 ml fresh water spilled on the bedding), and foreign object in the cage (an unusual novel plastic object). Anxiety and depression-like behaviors and maternal care were assessed about 1 week after delivery (Figure 1A). For EGCG treatment, freshly dissolved EGCG in saline was injected intraperitoneally daily for 5 days at a dose of 50 mg/kg (the first dose of EGCG was given on postpartum day 3). The EGCG was purchased from Sigma-Aldrich (St. Louis, MO, United States).

**Table 1:** The procedure of chronic unpredictable mild stress in pregnant mice during gestational day 7 (G7) to 16 (G16).

Day 1	Day 2	Day 3	Day 4	Day 5
Restraint stress 9 am–12 am	Fasting and water deprivation 9 am–3 pm	Restraint stress 11 am–1 pm	Cage tilt 10 am–5 pm	White noise 10 am–1 pm
White noise 3 pm–6 pm	Forced swim 5 pm	Tail pinch stimulation 3 pm		Forced swim 1 pm
Cage tilt 8 pm–8 am	Overnight lighting 7 pm–8 am	Paired housing 5 pm–10 pm	Soiled cage 7 pm–8 am	Foreign object in cage 5 pm–10 pm
Day 6	Day 7	Day 8	Day 9	Day 10
Tail pinch stimulation 10 pm	Paired housing 8 am–1 pm	Fasting and water deprivation 7 am–2 pm	Tail pinch stimulation 9 am	Cage tilt 7 am–3 pm
	Restraint stress 3 pm–6 pm	White noise 4 pm–7 pm	Forced swim 12 am	
Overnight lighting 7 pm–7 am	Cage tilt 8 pm–10 am	Soiled cage 9 pm–10 am	Foreign object in cage 3 pm–8 pm	Overnight lighting 5 pm–10 am

### 2.3. Offspring litter size and survival rate

The number of pups at birth was recorded. The survival rate of the offspring was calculated by dividing the number of pups on postnatal day 8 by the number of pups that were born.

### 2.4. Pup retrieval

Maternal behavior was evaluated based on pup retrieval (Gerecsei et al., 2018). Five days after parturition, pups were separated from the dam and placed at the opposite end of the cage. The female mouse was allowed 10 min to retrieve the pups and the latency to retrieval of the first and all other pups was recorded. The experiment was terminated when all pups were retrieved or after 10 min.

### 2.5. Open field test

A white opaque square box (45 × 45 × 45 cm) in a quiet room with dim lighting was used for the open field test (OFT). Each mouse was placed in the corner of the box and allowed 10 min to explore. The distance covered, amount of time spent in the center square, and number of entries into the center square were recorded.

### 2.6. Forced swimming test

The forced swimming test (FST) was carried out as previously described (Yang et al., 2019). Briefly, each mouse was placed in a transparent cylindrical container (diameter, 25 cm and height, 40 cm) filled with water to a height of 25 cm at a temperature of 25 ± 2°C, and was forced to swim in the container for 6 min. Immobility time during the last 4 min was recorded.

### 2.7. Tail suspension test

The tail suspension test (TST) was performed as previously described (Lu et al., 2021). Briefly, mice were suspended approximately 30 cm above the floor by tape placed about 1 cm from the tail tip. Each mouse was suspended for 6 min and immobility time during the test was recorded.

### 2.8. Immunofluorescence analysis

C-Fos expression in response to the FST was evaluated as a marker of neuronal activation (Rotllant et al., 2002; Ons et al., 2004). 90 min after the FST, mice were deeply anesthetized and intracardially perfused with phosphate-buffered saline followed by 4% paraformaldehyde solution. The whole brain was removed and postfixed overnight at 4°C in 4% paraformaldehyde before dehydration in 30% sucrose solution for at least 48 h. The brain was then serially sectioned at a thickness of 30 μm, and the sections were blocked for 2.5 h and incubated overnight at 4°C with c-Fos antibody (1:1000; Synaptic Systems, Göttingen, Germany) followed by incubation for 1 h at room temperature with secondary antibody (1:1000; Abcam, Cambridge, MA, United States). Images were acquired with a fluorescence microscope (Olympus, Tokyo, Japan; Model VS120) and analyzed with ImageJ software (NIH, Bethesda, MD, United States).

### 2.9. Real-time PCR

The hippocampus was dissected and frozen in liquid nitrogen and stored at −80°C. Total RNA was extracted with an RNA Isolation Kit (Vazyme, Shanghai, China) and reverse-transcribed to cDNA using reverse transcriptase (Vazyme, Shanghai, China). mRNA levels were quantified using SYBR qPCR Master Mix (Vazyme, Shanghai, China) using the primers shown in Table 2.

**Table 2:** The sequences of the primers used in this study.

Gene	Forward	Reverse
*sema3a*	TGTGCCAATTTCATCAAGGTCC	CTCTTCCCACGACCGTTTTCA
*gsk3β*	AAGCGATTTAAGAACCGAGAGC	AGAAATACCGCAGTCGGACTAT
*crmp2*	TCAAAGGTGGCAAGATTGTGAA	GGAATCACCATTCTGGAGTGG
*β-actin*	GTGACGTTGACATCCGTAAAGA	GCCGGACTCATCGTACTCC

### 2.10. Western blotting

Mice anesthetized with 2% pentobarbital sodium solution were sacrificed and the hippocampus was removed and homogenized in radioimmunoprecipitation assay buffer containing protease and phosphatase inhibitor. The homogenate was centrifuged at 13,200 rpm for 30 min at 4°C. The supernatant was collected and protein concentration was measured with a bicinchoninic acid protein assay kit (Thermo Fisher Scientific, Waltham, MA, United States). Protein samples were electrophoretically separated on 4–12% polyacrylamide gels and transferred to a polyvinylidene difluoride membrane (Bio-Rad, Hercules, CA, United States) that was blocked in 5% nonfat milk for 2 h at room temperature and incubated overnight at 4°C with primary antibodies against Sema3A (1:1000; Abcam, Cambridge, MA, United States) and phosphorylated (p-)GSK3β, GSK3β, p-CRMP2, CRMP2, and β-actin (all 1:1000 and from Cell Signaling Technology, Danvers, MA, United States). The membrane was then incubated with horseradish peroxidase-conjugated secondary antibody (1:1000; Cell Signaling Technology, Danvers, MA, United States) for 1 h at room temperature and immunoreactivity was analyzed using ImageJ.

### 2.11. Enzyme-linked immunosorbent assay

After behavioral tests, all mice were anesthetized with 2% pentobarbital sodium solution and blood was collected from the eyeballs. The whole blood was stored overnight at 4°C and then centrifuged at 5,000 rpm for 10 min at 4°C. The supernatant was stored at −80°C until use. Sema3A level was quantified with an enzyme-linked immunosorbent assay (ELISA) kit (CUSABIO, Wuhan, China) according to the manufacturer’s instructions.

### 2.12. Statistical analysis

Statistical analysis was performed using Prism v8.0 software (GraphPad, La Jolla, CA, United States). Data are presented as mean ± SEM. Comparisons were performed across 2 groups with the unpaired *t* test and across multiple groups by one-way analysis of variance followed by the Tukey *post hoc* test. Statistical differences were accepted as significant at *p* < 0.05.

## 3. Results

### 3.1. Gestational stress induces anxiety and depression-like behaviors during the postpartum period

**Figure 2:**
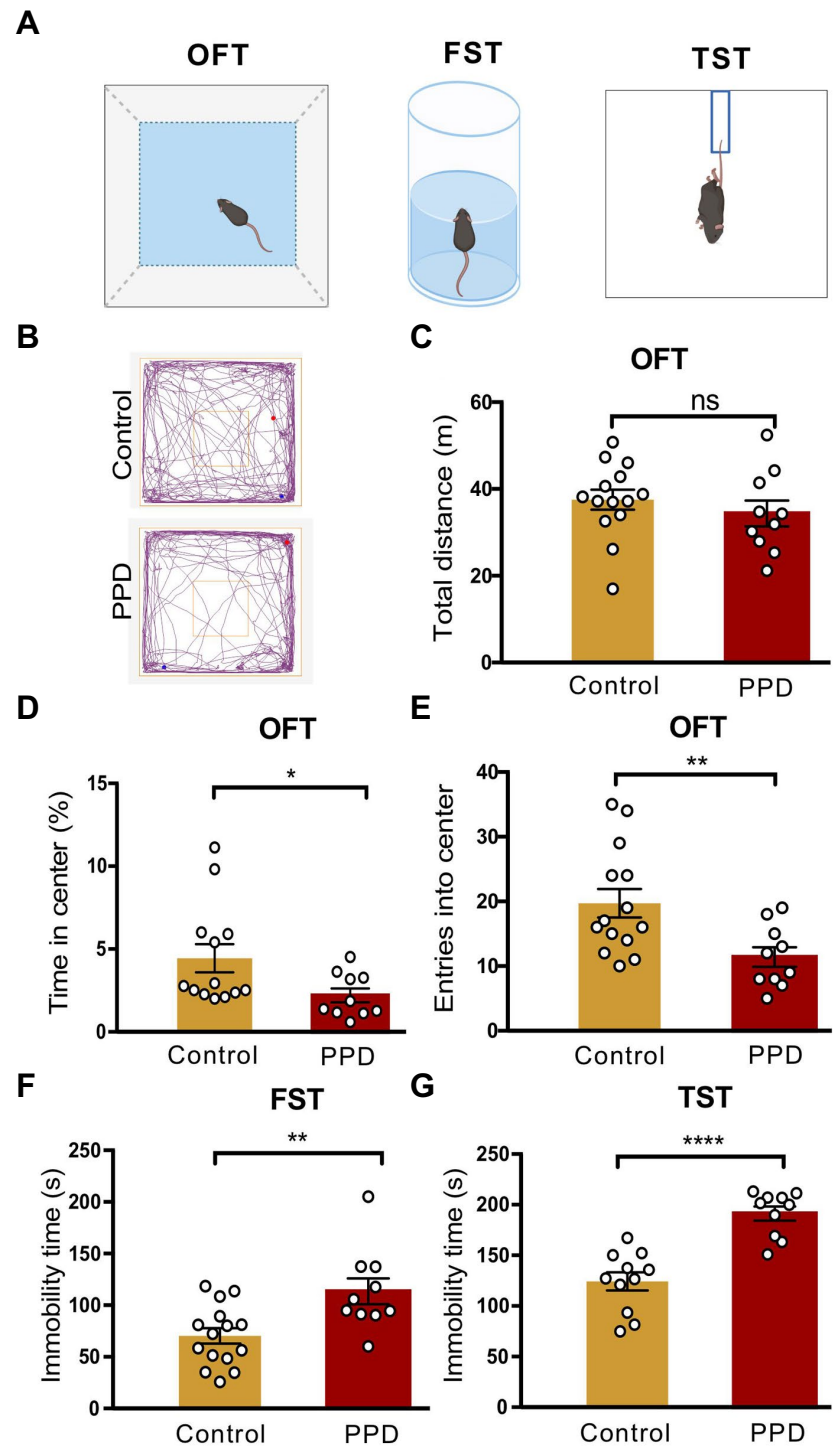
Gestational stress induced anxiety and depressive-like behaviors during the postpartum period. **(A)** The schematic diagram of the OFT, FST, and TST. **(B)** The representative movement tracks of each group. **(C–E)** Histograms showing the total distance traveled, time spent in the center, and number of entries into the center zone during the OFT. **(F)** Histogram showing the immobility time of mice in the FST. **(G)** Histogram showing the immobility time of mice in the TST. Data were mean ± SEM. White circles represent individual data points. *p < 0.05; **p < 0.01; ****p < 0.0001 vs. Con; ns, not significant (unpaired *t*-test).

Anxiety and depression-like behaviors in PPD model mice were evaluated with the OFT, FST, and TST (Figure 2A). Representative movement trajectories in the OFT for mice in each group were shown in Figure 2B. There was no significant difference in total distance traveled between PPD and control mice, suggesting that gestational stress had no effect on locomotion (*p* = 0.4010; Figure 2C). However, compared with control group, PPD mice spent less time in the center area (*p* < 0.05; Figure 2D) and had decreased entries into the center zone (*p* < 0.01; Figure 2E), suggesting that gestational stress caused postpartum anxiety-like behavior in mice. Gestational stress also increased immobility time in the FST (*p* < 0.01; Figure 2F) and TST (*p* < 0.0001; Figure 2G), indicating that chronic unpredictable mild stress during gestation induced postpartum depression-like behavior in mice.

Depressed mothers exhibit significantly more negative behaviors and disengagement toward their children compared with those without depression (Lovejoy et al., 2000). To determine whether PPD had a detrimental effect on maternal care and pup development, we recorded the litter size at birth and pup survival rate on postpartum day 8, as well as the maternal behaviors of the dam. However, there were no significant differences in offspring litter size (*p* = 0.8639; Supplementary Figure 1A), survival rate (*p* = 0.3243; Supplementary Figure 1B), or pup retrieval including latency to retrieve the first pup (*p* = 0.9408; Supplementary Figure 1C) and all pups (*p* = 0.1078; Supplementary Figure 1D) between the PPD and control groups.

### 3.2. Gestational stress decreases c-Fos expression in the hippocampus

**Figure 3:**
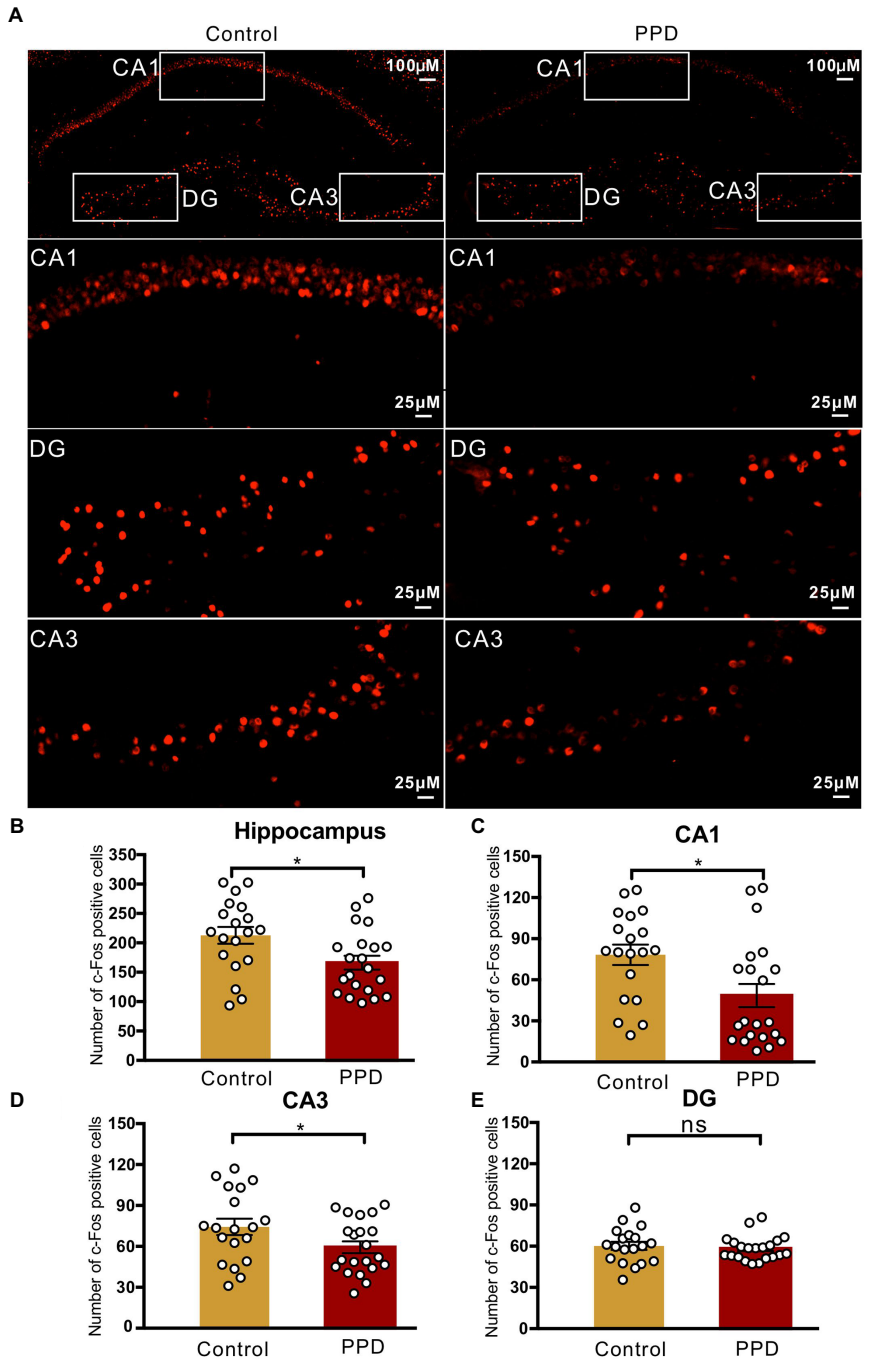
c-Fos positive cells in the hippocampus. **(A)** Representative images of c-Fos (red) positive cells in the hippocampus. **(B)** The number of c-Fos positive cells in the hippocampus. **(C–E)** The number of c-Fos positive cells in the subregions of the CA1 **(C)**, CA3 **(D)**, and DG **(E)**. Data were mean ± SEM. White circles represent individual data points. *p < 0.05 vs. Con; ns, not significant (unpaired *t*-test). CA, cornu ammonis; DG, dentate gyrus.

To determine the involvement of the hippocampus in gestational stress-induced anxiety and depression-like behaviors, we performed immunofluorescence labeling of c-Fos after the FST (Figure 3A). The number of c-Fos–positive cells in the hippocampus was significantly decreased in the PPD group compared with the control group (*p* < 0.05; Figure 3B), suggesting that decreased activation of the hippocampus was related to gestational stress-induced anxiety and depression-like behaviors. Specifically, gestational stress caused a marked reduction in c-Fos levels in the CA1 (*p* < 0.05; Figure 3C) and CA3 (*p* < 0.05; Figure 3D) areas while having no effect on the c-Fos level in the dentate gyrus (*p* = 0.5882; Figure 3E).

### 3.3. Gestational stress enhances Sema3A expression and inhibits GSK3β phosphorylation in the hippocampus

**Figure 4:**
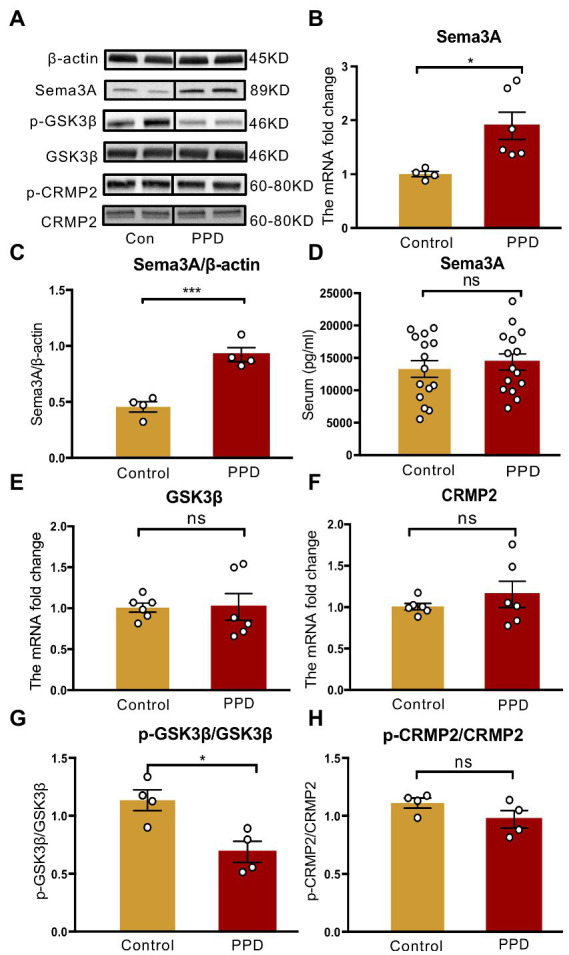
Gestational stress enhanced the hippocampal expression of Sema3A and inhibited the phosphorylation of GSK3β. **(A)** Representative western blot images indicating the Sema3A, GSK3β, p-GSK3β, CRMP2, p-CRMP2, and β-Actin protein bands. Full-length blots are presented in Supplementary Figure 2. **(B,C)** The mRNA and protein expressions of Sema3A in two groups. **(D)** The level of serum Sema3A examined by ELISA in two groups. **(E,F)** The mRNA expression of GSK3β, CRMP2 in two groups. **(G,H)** The relative protein expression of p-GSK3β/GSK3β and p-CRMP2/CRMP2 and in two groups. Data were mean ± SEM. White circles represent individual data points. *p < 0.05; ***p < 0.001 vs. Con; ns, not significant (unpaired *t*-test).

An elevated level of Sema3A in the central nervous system (CNS) including the hippocampus and cerebellum has been observed in various neurologic and psychiatric diseases (Eastwood et al., 2003; Good et al., 2004; Van Battum et al., 2015), but its role in depression is not fully understood. We examined Sema3A expression in the hippocampus and peripheral blood and found that the mRNA (*p* < 0.05; Figure 4B) and protein (*p* < 0.001; Figures 4A,C) levels in the hippocampus were significantly higher in the PPD group than the control group. However, there was no difference in serum Sema3A level between groups (*p* = 0.5537, Figure 4D).

As GSK3β and CRMP2 were linked to major depression, we examined the mRNA and protein levels of GSK3β and CRMP2 in the hippocampus. Compared with the control group, there were no significant changes in GSK3β (*p* = 0.9569; Figure 4E) and CRMP2 (*p* = 0.3859; Figure 4F) transcript levels in PPD mice; however, gestational stress decreased the ratio of p-GSK3β to GSK3β (*p* < 0.05 vs. control; Figures 4A,G) in the hippocampus, indicating an increase in the expression of active GSK3β. There was no difference in phosphorylated CRMP2 level in the hippocampus between groups (*p* = 0.1551; Figures 4A,H).

### 3.4. EGCG alleviates postpartum anxiety and depression-like behaviors in PPD mice

**Figure 5:**
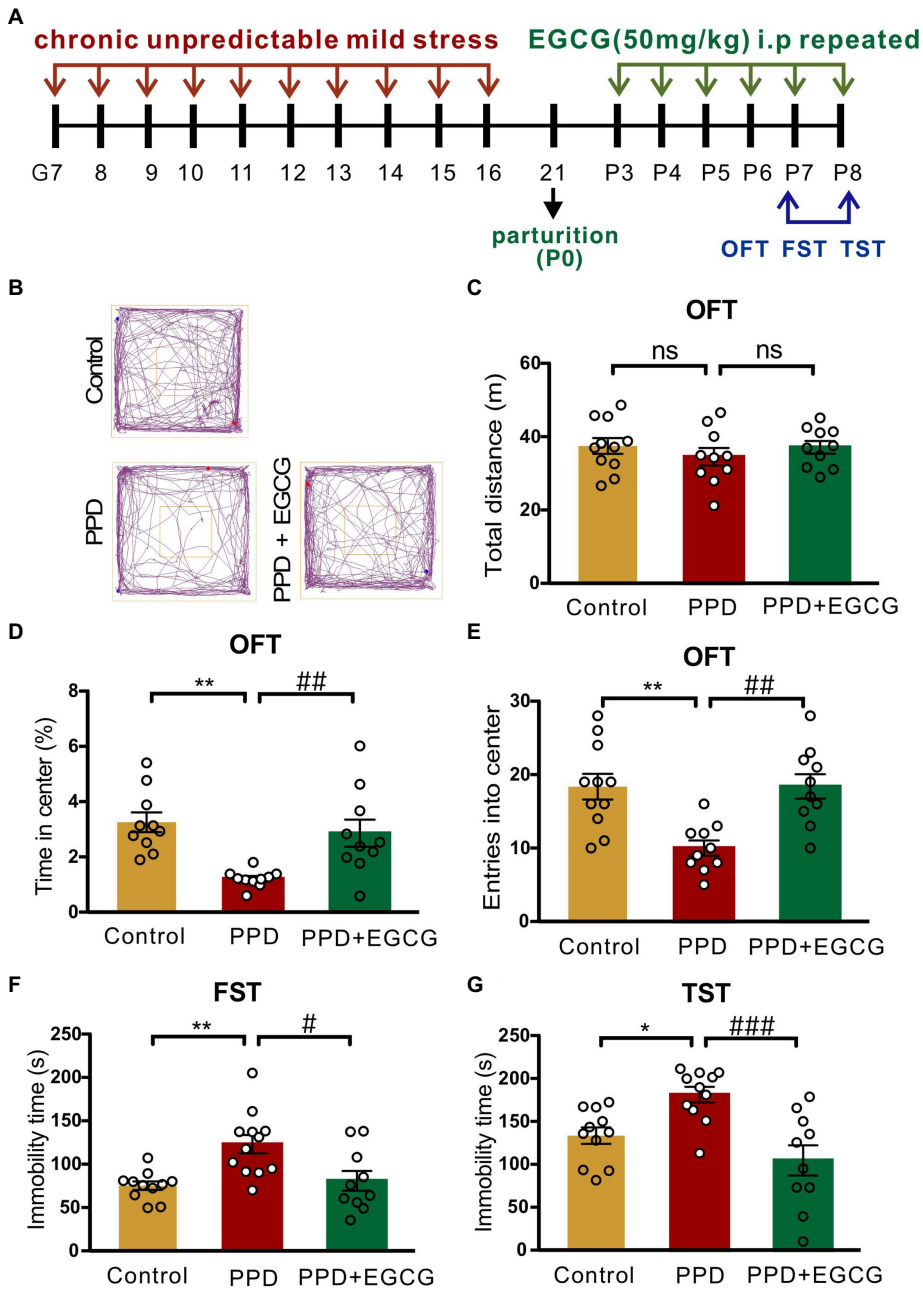
EGCG alleviated the postpartum anxiety and depressive-like behaviors of PPD-mice. **(A)** Experimental diagram showing the timeline of model establishment, drug administration and behavioral tests. **(B)** The representative movement tracks of each group. **(C–E)** Histograms showing the total distance traveled, time spent in the center, and number of entries into the center of mice during the OFT. **(F)** Histogram showing the immobility time of mice in the FST. **(G)** Histogram showing the immobility time of mice in the TST. Data were mean ± SEM. White circles represent individual data points. *p < 0.05; **p < 0.01 vs. Con; ^#^*p* < 0.05; ^##^*p* < 0.01, ^###^*p* < 0.001 vs. PPD; ns, not significant.

As EGCG had anti-inflammatory, antioxidative, and neuroprotective effects in mammals (Chakrawarti et al., 2016), we investigated whether it could alleviate the anxiety and depression-like behaviors associated with PPD. The experimental timelines of drug administration and behavioral testing were shown in Figure 5A. Representative movement trajectories of mice from each group in the OFT were shown in Figure 5B. There was no difference across groups in total distance covered [*F*_(2,28)_ = 0.5797, *p* = 0.5666; Figure 5C], but there were significant differences in the time spent in the center area [*F*_(2,27)_ = 9.336, *p* < 0.001; Figure 5D] and entries into the center zone [*F*_(2,28)_ = 9.697, *p* < 0.001; Figure 5E]. Specifically, mice exposed to gestational stress spent less time in the center area (*p* < 0.01; Figure 5D) and exhibited fewer entries into the center zone (*p* < 0.01; Figure 5E) than controls; EGCG treatment abrogated these decreases (time in center area: *p* < 0.01, Figure 5D; center zone entries: *p* < 0.01, Figure 5E). There was also a significant difference in immobility time in the FST [*F*_(2,30)_ = 8.045, *p* < 0.01; Figure 5F] and TST [*F*_(2,29)_ = 9.735, *p* < 0.001; Figure 5G] across groups. Multiple comparisons revealed that mice in the PPD group had longer immobility time in both tests (FST: *p* < 0.01, Figure 5F; TST: *p* < 0.05, Figure 5G) than control mice. EGCG decreased immobility time in the FST (*p* < 0.05; Figure 5F) and TST (*p* < 0.001; Figure 5G) in mice exposed to gestational stress.

### 3.5. EGCG inhibits Sema3A expression and promotes GSK3β phosphorylation in the hippocampus

**Figure 6:**
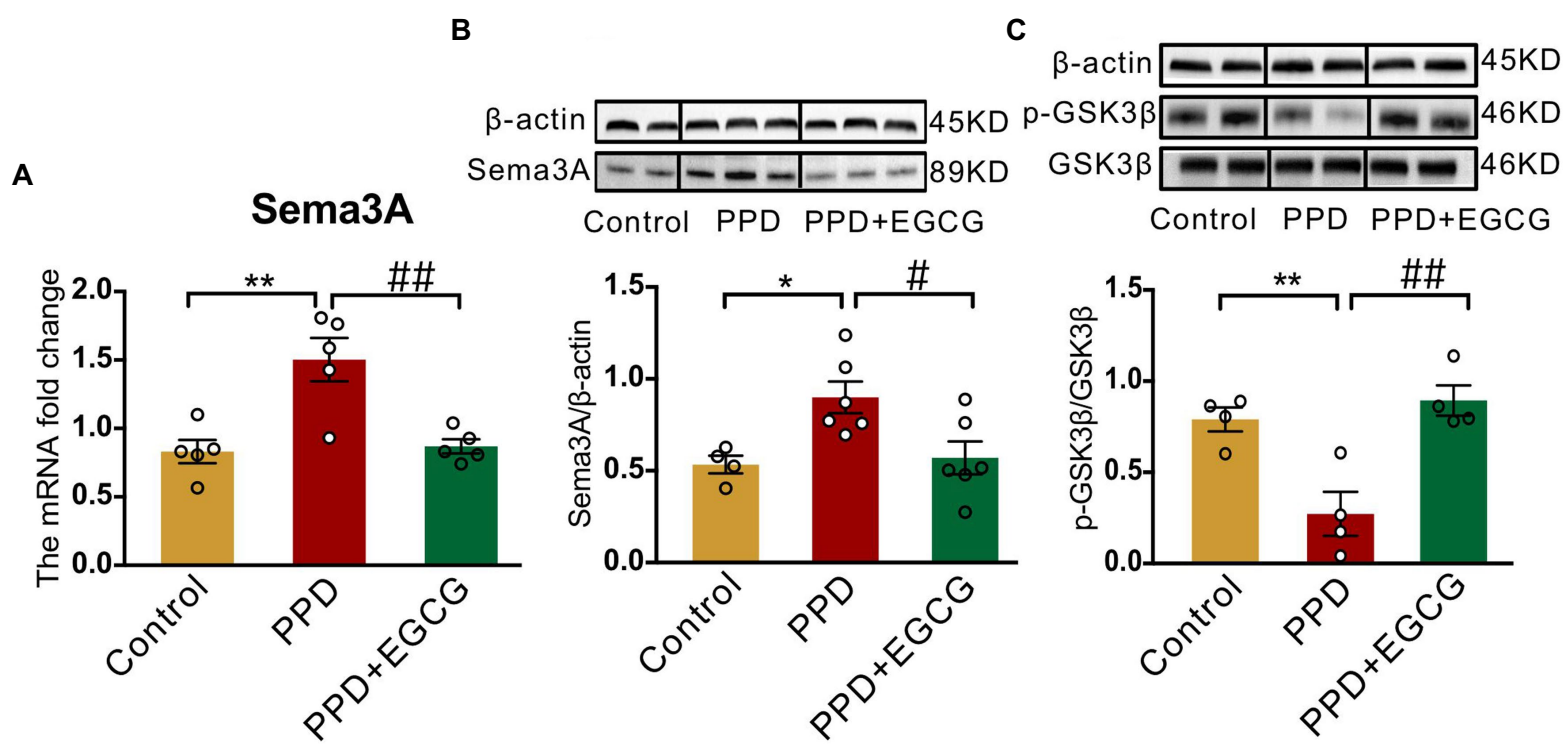
EGCG inhibited the hippocampal expression of Sema3A and promoted GSK3β phosphorylation. **(A)** The Sema3A mRNA level in the hippocampus of each group. The protein levels of Sema3A **(B)** and p-GSK3β **(C)** in the hippocampus of the three groups. Full-length blots are presented in Supplementary Figure 3. Data were mean ± SEM. White circles represent individual data points. *p < 0.05; **p < 0.01 vs. Con; ^#^*p* < 0.05; ^##^*p* < 0.01 vs. PPD.

To clarify the molecular basis for the effects of EGCG in mice with PPD, we examined the expression of Sema3A, p-GSK3β, and GSK3β in the hippocampus. There were significant differences among groups in Sema3A mRNA [*F*_(2,12)_ = 12.25, *p* < 0.01; Figure 6A] and protein [*F*_(2,13)_ = 5.942, *p* < 0.05; Figure 6B] levels as well as p-GSK3β/GSK3β protein ratio [*F*_(2,9)_ = 12.91, *p* < 0.01; Figure 6C] in the hippocampus. Sema3A mRNA (PPD vs. PPD + EGCG, *p* < 0.01; Figure 6A) and protein (PPD vs. PPD + EGCG, *p* < 0.05; Figure 6B) expression was decreased by EGCG treatment, whereas the ratio of p-GSK3β/GSK3β was increased (PPD vs. PPD + EGCG, *p* < 0.01; Figure 6C), indicating a reduction of active GSK3β in the hippocampus by EGCG treatment. These results suggest that EGCG mitigates anxiety and depression-like symptoms in PPD mice by inhibiting Sema3A and GSK3β signaling.

## 4. Discussion

**Figure 7:**
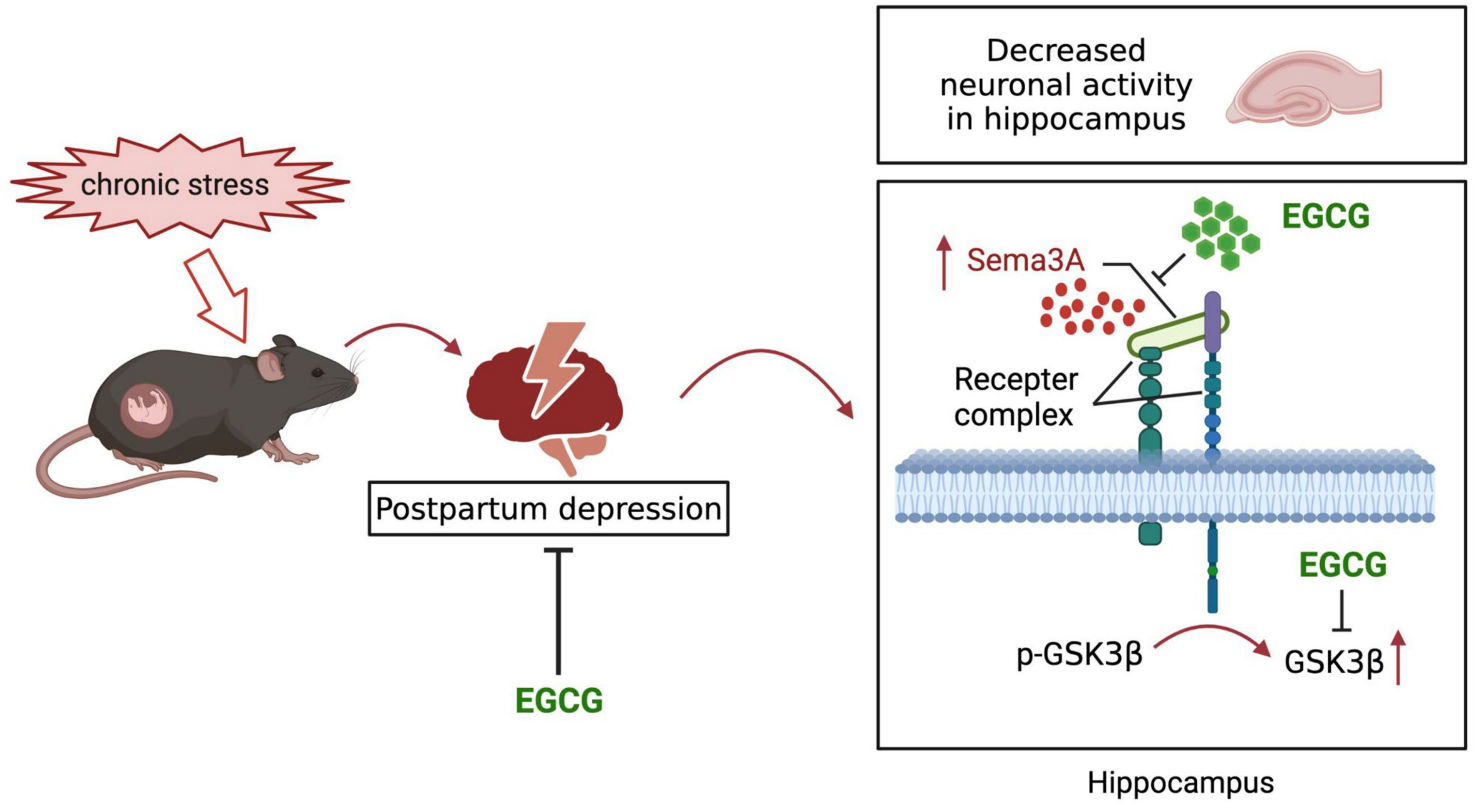
Schematic diagram of the potential mechanisms of EGCG against the anxiety and depression-like behaviors in postpartum period induced by gestational stress.

EGCG, an abundant catechin in tea, exhibited protective effect on depression, but its effect on postpartum anxiety and depressive symptoms induced by gestational stress remained unclear. In this study, we found that the postpartum anxiety and depressive symptoms, as well as the increased expression of hippocampal Sema3A and decreased phosphorylation of GSK3β caused by gestational stress could be reversed by systemic administration EGCG (Figure 7).

An elevated level of Sema3A in the CNS was linked to various psychiatric disorders (Van Battum et al., 2015) and *Sema3A* risk variants were related to major depression (Zhou et al., 2017). In the present study, we found that Sema3A expression in the hippocampus was increased in a mouse model of PPD, which was associated with anxiety and depression-like symptoms. As Sema3A is an axon guidance molecule (Perez et al., 2021) and the accumulation of Sema3A in the hippocampus can induce programmed cell death of neurons (Good et al., 2004), we speculate that the upregulation of hippocampal Sema3A in PPD mice influences synaptic plasticity or neuronal activity to cause anxiety and depression-like behaviors.

EGCG was shown to alleviate anxiety-like behavior and neuroinflammation in male rats with myocardial infarction (Wang et al., 2020) as well as CUMS-induced depression symptoms in male rats (Li G. et al., 2020). *In vivo* and *in vitro* experiments have shown that EGCG inhibited the expression of Sema3A and attenuated inflammation and apoptosis in rodents with lipopolysaccharide-induced acute kidney injury (Tian et al., 2018). In the present work, treatment with EGCG reduced anxiety and depression-like behaviors in PPD mice and reversed the increase of hippocampal Sema3A expression induced by gestational stress, indicating that it had protective effects against anxiety and depression in the postpartum period.

As a downstream target of Sema3A, GSK3β is thought to be associated with major depression. An increase in the activity of GSK3β has been observed in the prefrontal cortex of patients and suicide victims with depression (Karege et al., 2007), as well as in the platelets of patients with depression (Diniz et al., 2011). Animal studies have also provided evidence for the involvement of GSK3β in depression. GSK3α/β knock-in mice showed increased stress-induced depression-like behaviors (Polter et al., 2010), while inhibiting the activity of GSK3β reduced depression symptoms (Garza et al., 2012). Downregulation of Ser9-phosphorylated GSK3β in the nucleus accumbens was observed in a mouse social defeat model of depression; meanwhile, increasing the expression of inactive GSK3β enhanced resilience to social defeat stress (Wilkinson et al., 2011). Consistent with previous studies, we found that Ser9 phosphorylation of GSK3β was reduced in the hippocampus of PPD mice, suggesting that the increased activity of GSK3β contributed to the anxiety and depression-like behaviors induced by gestational stress. It is suggested that active GSK3β is strongly associated with the downscaling of synapses, declined neuronal excitability and increased inflammatory response (Duda et al., 2020), so we speculate that the increased GSK3β activity may be involved in PPD by affecting neuroplasticity and neuroinflammation. Protein kinase B (AKT)/GSK3β/CRMP2 signaling was shown to be involved in the development of depression induced by stress (Wei et al., 2021). In a rat model of Parkinson disease, EGCG promoted the phosphorylation of AKT and GSK3β and reduced neuron apoptosis in the substantia nigra (Zhou et al., 2019). The results of *in vitro* experiments showed that EGCG enhanced the survival of A549 cells *via* activation of AKT (Kim et al., 2009). We found that EGCG treatment increased GSK3β phosphorylation, implying that it could alleviate PPD symptoms by inhibiting GSK3β activity in the hippocampus.

CRMP2, a phosphorylation target of GSK3β, has been implicated in various models of depression induced by stress (Szego et al., 2010; Yang et al., 2014). CRMP2 regulates various aspects of neuronal development, including axon guidance, dendritic morphogenesis and synaptic plasticity (Zhang and Koch, 2017). The upregulation of phosphorylated CRMP2 results in the impairment of neuronal plasticity and neural function (Quach et al., 2015), which relate to the development of depression. Contrary to our expectation, we found that gestational stress did not affect the expression or phosphorylation of CRMP2 in the hippocampus. This may be because gestational stress affects the function but not the expression of CRMP2 or because another downstream effector of GSK3β mediates the anxiety and depression-like symptoms observed in PPD; additional studies are needed to investigate these possibilities.

In conclusion, we demonstrated for the first time that PPD is associated with elevated expression of Sema3A and decreased phosphorylation of GSK3β in the hippocampus. We also found that EGCG improved PPD *via* a mechanism involving the downregulation of Sema3A and increased GSK3β phosphorylation in the hippocampus. These results suggest that EGCG has therapeutic potential for the treatment of PPD.

## Data availability statement

The original contributions presented in the study are included in the article/Supplementary material, further inquiries can be directed to the corresponding author.

## Ethics statement

The animal study was reviewed and approved by the Animal Ethics and Welfare Committee of Zhejiang University School of Medicine (Ethics code: ZJU20210216).

## Author contributions

XC and FX designed and conceptualized the work. FX, HW, LX, QX, and QC generated the data and drafted the manuscript. FX, LS, HL, and JX contributed to the interpretation of data. XC, FX, and HW reviewed and edited the manuscript. All authors read and approved the final manuscript.

## Funding

This study was supported by funding from the National Natural Science Foundation of China (NSFC, Nos. 81271237, 81471126, and 81501702).

## Conflict of interest

The authors declare that the research was conducted in the absence of any commercial or financial relationships that could be construed as a potential conflict of interest.

The handling editor HZ declared a shared parent affiliation with the authors at the time of review.

## Publisher’s note

All claims expressed in this article are solely those of the authors and do not necessarily represent those of their affiliated organizations, or those of the publisher, the editors and the reviewers. Any product that may be evaluated in this article, or claim that may be made by its manufacturer, is not guaranteed or endorsed by the publisher.

## Supplementary material

SUPPLEMENTARY FIGURE 1 The effect of gestational stress on maternal care of postpartum mice. **(A)** Quantitative results of litter size. **(B)** The survival rate of the offspring. **(C,D)** Histograms showing the time to retrieve the first pup and all pups in a pup retrieval task. Data were mean ± SEM. White circles represent individual data points. ns, not significant (unpaired *t*-test).

SUPPLEMENTAL FIGURE 2 Full-length blots about western blot of Figure 4.

SUPPLEMENTAL FIGURE 3 Full-length blots about western blot of Figure 6.
