# Supplementary figures and images for: Epigallocatechin-3-gallate alleviates gestational stress-induced postpartum anxiety and depression-like behaviors in mice by downregulating semaphorin3A and promoting GSK3β phosphorylation in the hippocampus
Source: Front Mol Neurosci. 2023 Jan 26;15:1109458. doi: 10.3389/fnmol.2022.1109458 (PMC9909483; doi:10.3389/fnmol.2022.1109458)

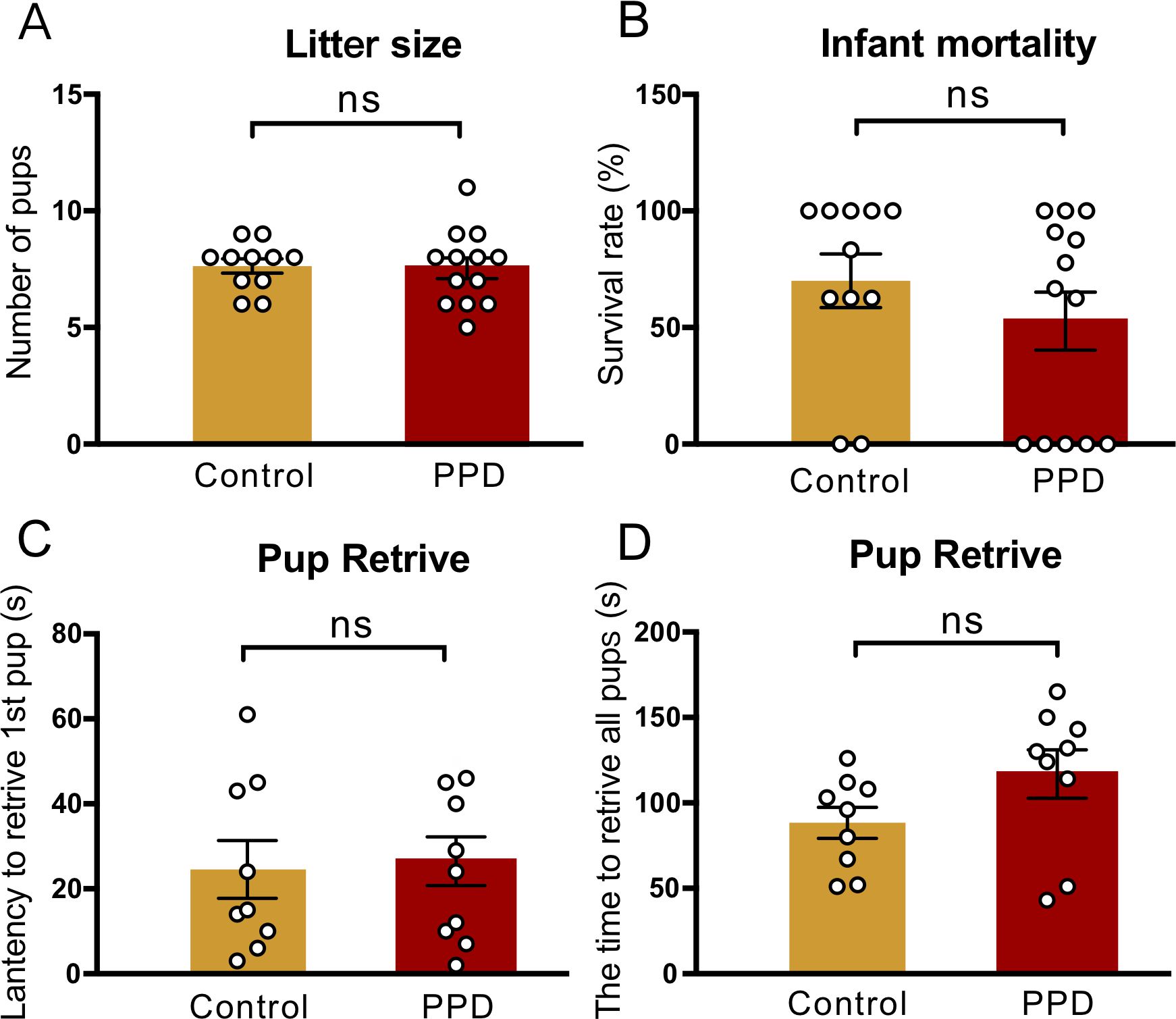

Supplement: SUPPLEMENTARY FIGURE 1 — The effect of gestational stress on maternal care of postpartum mice. (A) Quantitative results of litter size. (B) The survival rate of the offspring. (C,D) Histograms showing the time to retrieve the first pup and all pups in a pup retrieval task. Data were mean ± SEM. White circles represent individual data points. ns, not significant (unpaired t-test). [file Image_1.jpg]

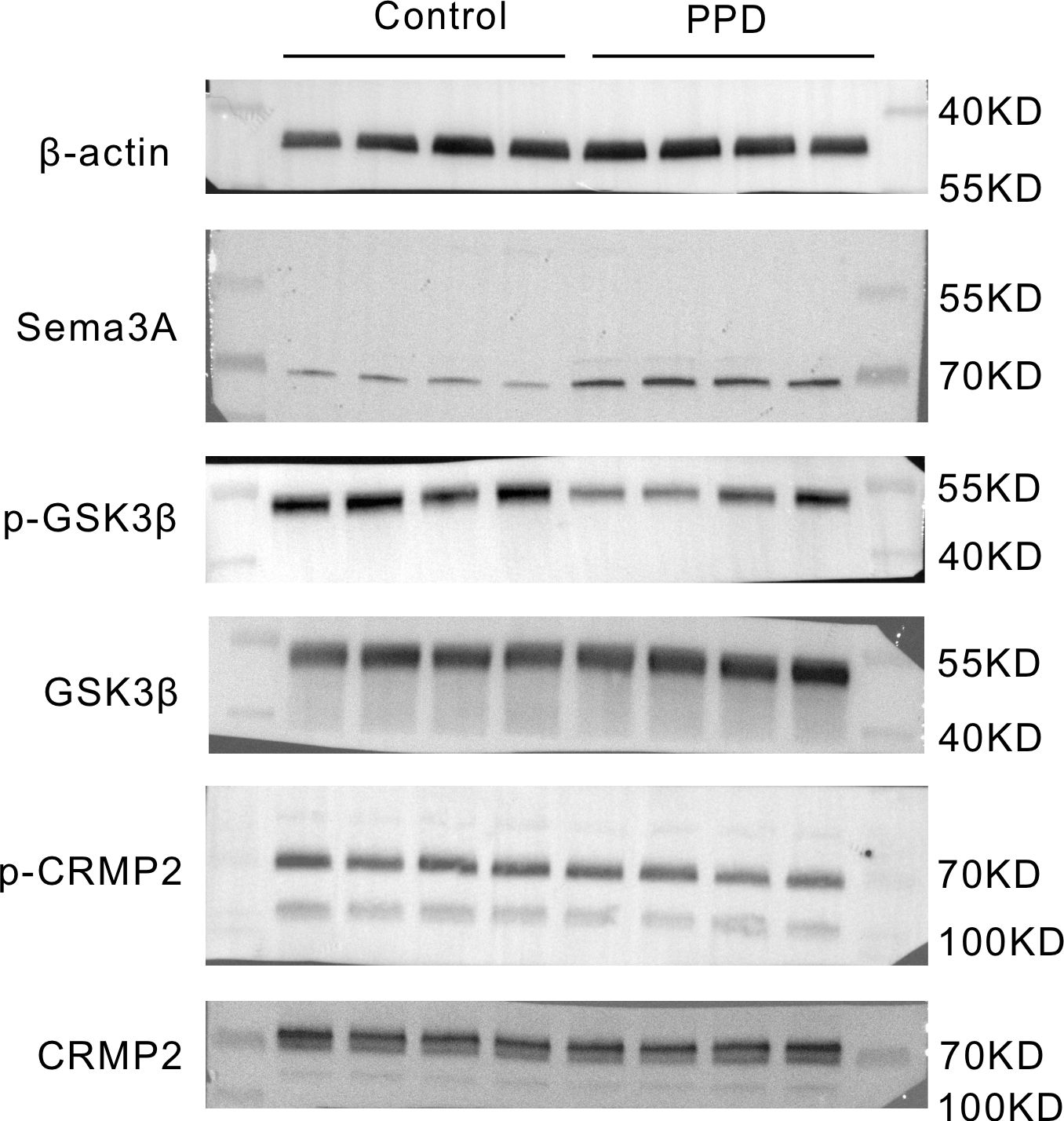

Supplement: SUPPLEMENTAL FIGURE 2 — Full-length blots about western blot of Figure 4. [file Image_2.JPEG]

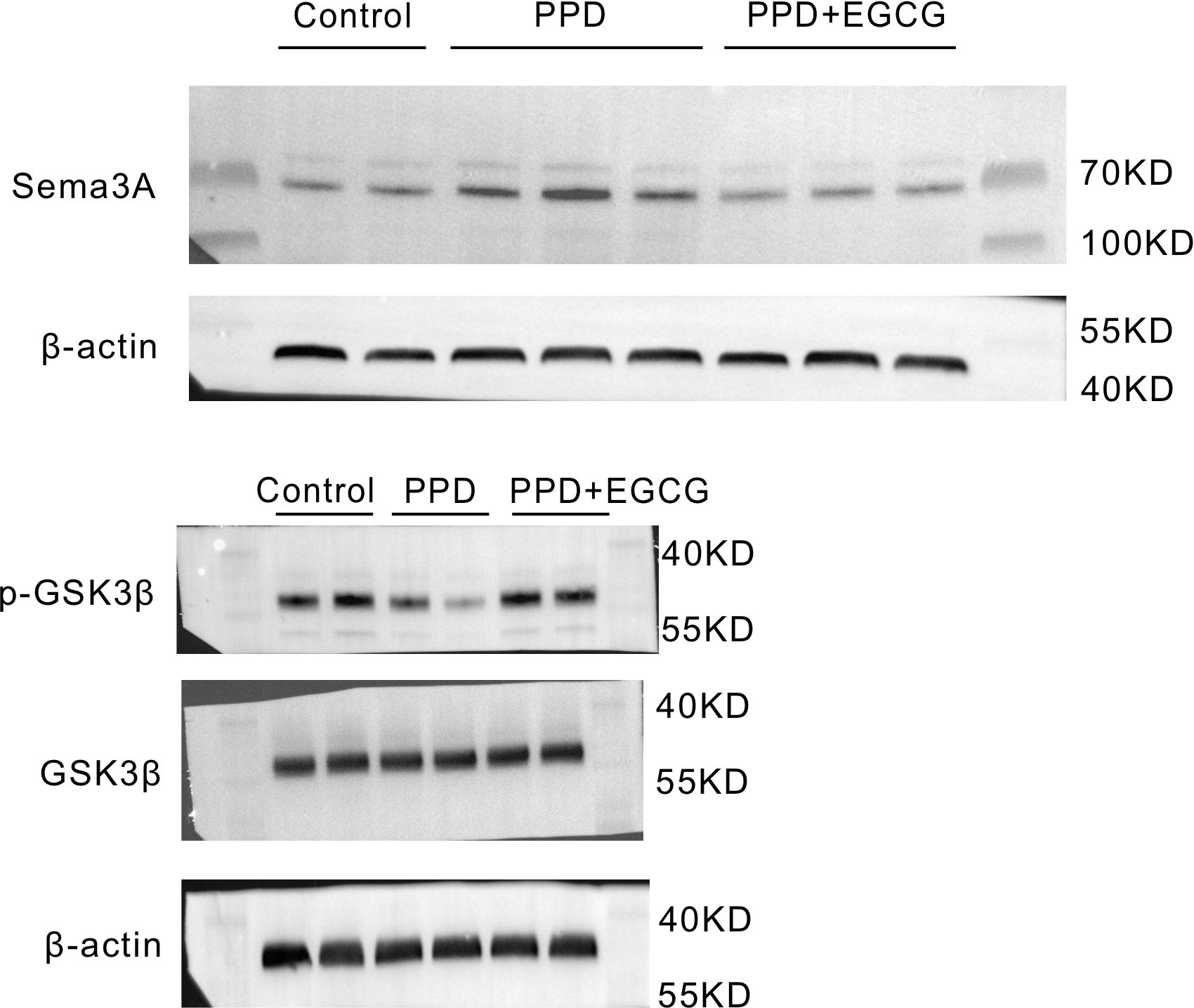

Supplement: SUPPLEMENTAL FIGURE 3 — Full-length blots about western blot of Figure 6. [file Image_3.JPEG]
